# North American diadromous fishes: Drivers of decline and potential for recovery in the Anthropocene

**DOI:** 10.1126/sciadv.abl5486

**Published:** 2022-01-28

**Authors:** John R. Waldman, Thomas P. Quinn

**Affiliations:** 1 Queens College and Graduate School, City University of New York, New York, NY, USA.; 2 University of Washington, Seattle, WA, USA.

## Abstract

Diadromous fishes migrate between freshwater and marine habitats to complete their life cycle, a complexity that makes them vulnerable to the adverse effects of current and past human activities on land and in the oceans. Many North American species are critically endangered, and entire populations have been lost. Major factors driving declines include overfishing, pollution, water withdrawals, aquaculture, non-native species, habitat degradation, over-zealous application of hatcheries designed to mitigate effects of other factors, and effects of climate change. Perhaps, the most broadly tractable and effective factors affecting diadromous fishes are removals of the dams that prevent or hinder their migrations, alter their environment, and often favor non-native biotic communities. Future survival of many diadromous fish populations may depend on this.

## Teaser

Many drivers have reduced North American diadromous fishes, but dam removal offers the most viable restoration option.

## INTRODUCTION

Environmental problems that involve coupled human and natural systems have long and complex histories, commonly resulting in muddled and ineffective prospects for remediation. North America’s diadromous fish species (a term that includes anadromous species, spawned in freshwater habitats and migrating to sea to feed before returning to fresh water to spawn, and catadromous species following the reverse pathway) entered into the Anthropocene having suffered among the most marked declines of any vertebrate taxon on the continent. These declines have occurred despite the seemingly inexhaustible abundance of these fishes in Colonial times, on the basis of accounts of early European observers (*1*), and their continuing and widely recognized importance to humans and natural ecosystems.

In 1728, William Byrd II wrote of river herring (*Alosa* spp.) in a Virginia river “In a word, it is unbelievable, indeed, indescribable, as also incomprehensible, what quantity is found. One must behold oneself.” Some rivers were so filled with the flashing bodies of migrating fish that they were said to be “running silver” (*1*). Similar reports were common with Atlantic and Pacific salmon. For example, Montgomery (*2*) quoted Ezra Meeker in 1921 describing “…salmon so numerous in the shoal water of the [Puyallup River] as to literally touch each other” and the Lewis and Clark expedition commenting that in the Columbia River “… the multitude of [salmon] is almost inconceivable.”

Diadromous fishes of North America were once a major food of great economic value but, today, have been extirpated from much of their natural habitat. Ecologically, they are important as vectors for nutrient exchanges between rivers, estuaries, and marine waters. A variety of organisms feed on them, ranging from brown bears to birds to scavenging insects and microbes, benefiting from these nutrients in direct and indirect ways (*3*). Most notably, Pacific salmon (genus *Oncorhynchus*) die after spawning and contribute large inputs of marine-derived nutrients to watersheds (*4*, *5*), and lesser effects of other diadromous fishes have also been reported (*6*, *7*). These fishes are also important as prey in marine food webs. For example, the reduction in juvenile river herring as prey in the Gulf of Maine was linked to the loss of inshore Atlantic cod (*Gadus morhua*) and other gadids (*8*). However, many sharply reduced populations of diadromous fishes persist but no longer serve their original ecological role, sometimes termed as “ghost species” (*1*).

**Fig. 1.**
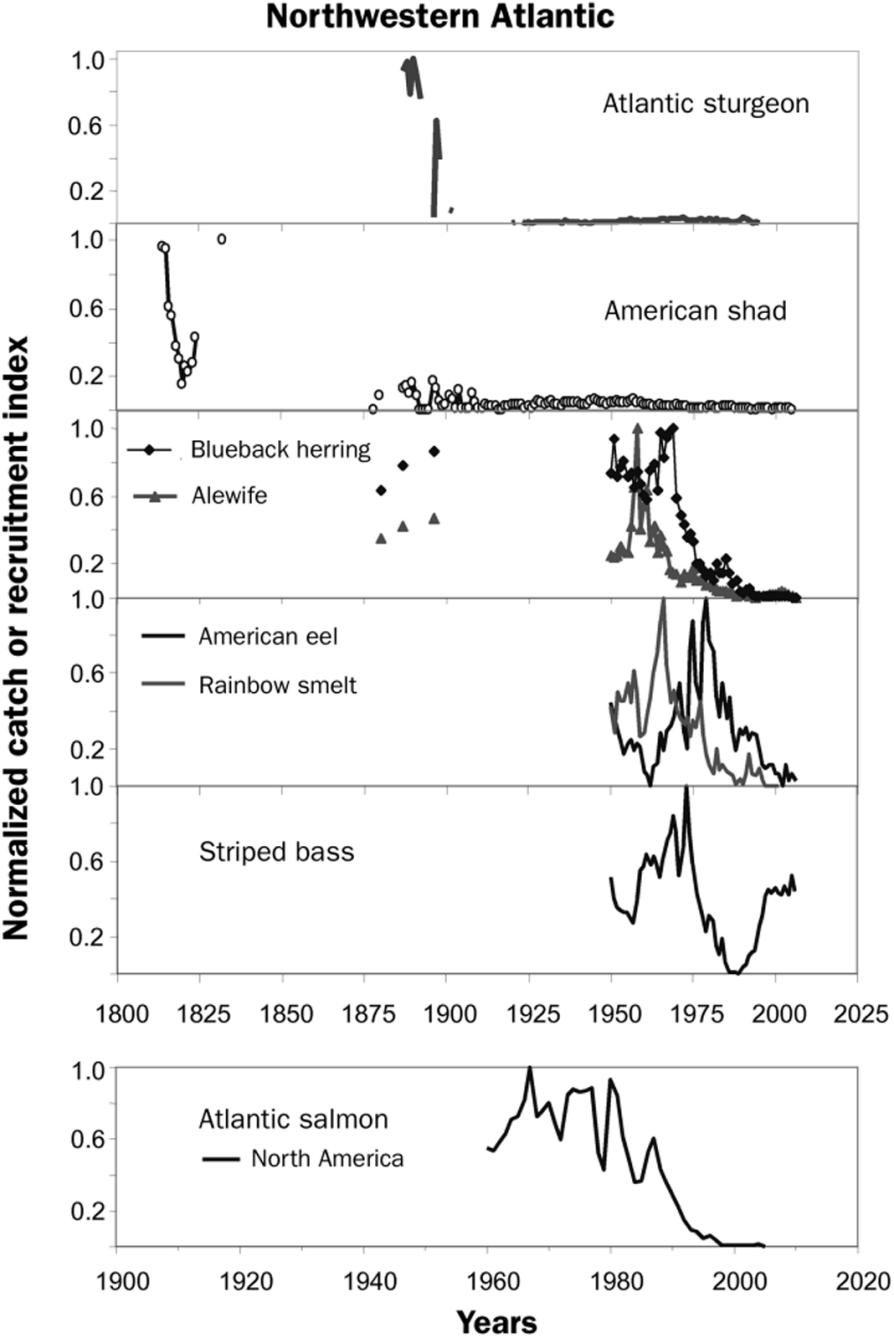
Normalized time series of indices of abundance of selected North Atlantic diadromous species. Only striped bass has shown a recovery. Data are U.S. summary statistics (*16*). Table adapted from Limburg and Waldman (*16*).

The current depleted state of these fishes (e.g., U.S. Atlantic Coast; Fig. 1) in many regions contrasts with their historically high societal values; most were deeply interwoven into regional sustenance and cultural practices of indigenous people and settlers from abroad. Because the spawning migrations of anadromous fishes often place them within easy reach of humans, many of these runs have been particularly important food sources. American shad (*Alosa sapidissima*) were a staple of residents along major mid-Atlantic rivers, where their spring arrival was celebrated with community “shad bakes” (*1*). Native Americans, including an “Eel Clan,” made great use of American eels (*Anguilla rostrata*) for food and other purposes. Eels may have comprised as much as 40% of the fish biomass in the Lake Ontario watershed (*9*). Migratory striped bass (*Morone saxatilis*) are the premier recreational inshore gamefish from Maryland through Quebec and supported commercial fisheries that landed as much 6700 metric tons along the Mid-Atlantic Coast in 1973 (*10*). Some populations of Atlantic sturgeon (*Acipenser oxyrinchus*), particularly in the Delaware River, were nearly extirpated by an intensive fishery in the late 1800s as part of a worldwide “caviar craze” (*11*).

Pacific salmon played a key role in the culture and sustenance of indigenous peoples around the Pacific Rim, from Japan, Russia, Alaska, and south to California (*12*, *13*), and they are the most valuable fin-fish resource in the United States (U.S. Department of Commerce data). A smaller anadromous species, the eulachon (*Thaleichthys pacificus*), was also important to indigenous peoples. These fish are commonly known as candlefish because their oil content is so high that they can be dried, fitted with a wick, and burned like a candle. On the U.S. and Canadian West Coast, Pacific lamprey (*Lampetra tridentata*) are of cultural significance for ceremonial, subsistence, and medicinal uses (*14*). Thus, there has been considerable reliance on a variety of diadromous fishes on both coasts of North America.

The losses of diadromous fishes have manifested in two ways. First, many entire populations have been extirpated. On the East Coast, 68 of the original 138 populations of American shad were lost (*15*), along with one-third of the Atlantic salmon (*Salmo salar*) populations (*16*). Only one of the original two populations of the glacial relic Atlantic whitefish (*Coregonus huntsmani*) in Nova Scotia persists, and it has been purposely landlocked to prevent losses in its unprotected marine range (*16*). Some species have lost populations incrementally at the southern extremes of their ranges: rainbow smelt (*Osmerus mordax*) from the Chesapeake Bay through Connecticut and alewife (*Alosa pseudoharengus*) in South Carolina. Alabama shad (*Alosa alabamae*) once ran in 59 rivers that flow into the Gulf of Mexico; today, they are known from 21, often in relict numbers (*17*).

On the Pacific Coast, through the late 1980s, fisheries managers lacked synoptic information on the status of salmon and trout over large areas. The reductions and losses of local populations went largely unnoticed until Nehlsen *et al.* (*18*) investigated and issued a clarion call on the numbers of stocks already extinct or in jeopardy. Their crude aggregation of salmon and trout “stocks at risk” numbered 214 from Washington, Oregon, Idaho, and California, with 101 at high risk of extinction, 58 at moderate risk, 54 of special concern, and 1 already listed under the U.S. Endangered Species Act (ESA). The authors noted the “records of extinct salmon and steelhead (*Oncorhynchus mykiss*) populations along the West Coast are sketchy” but concluded that at least 106 major populations had been extirpated. This report was soon followed by assessments by state and provincial entities on the status of salmon populations under their jurisdictions. Expectedly, the regions farther north, where human influences are less pronounced, had not only more populations in healthy condition but also greater uncertainty because various populations were not monitored adequately.

More common than extirpations are extreme reductions in abundances. In a status review of diadromous fish populations in North America and Europe, relative abundances had dropped more than 98% from historic highs in 13 of 35 time series and more than 90% in an additional 11 (*16*). Furthermore, most, such as American shad, had reached their lowest levels near the end of the observation period (*16*). Pacific lamprey numbers have declined greatly; e.g., counts at Winchester Dam located in the coastal Umpqua River decreased exponentially from a maximum of 46,785 in 1966 to 34 individuals in 2001 (*19*). There have been many reviews of these losses of populations and reductions in Pacific salmon abundance over broad spatial scales (*20*) and specific basins or regions (*21*, *22*). For example, in California, coho salmon (*Oncorhynchus kisutch*) ran in the hundreds of thousands just 60 years ago and were important members of the state’s coastal stream and ocean ecosystems (*23*), but recently, they number in the hundreds (*24*).

These declines are manifested in the species’ conservation statuses. Both Atlantic sturgeon and shortnose sturgeon (*Acipenser brevirostrum*) are on the U.S. ESA list, as are most American populations of Atlantic salmon. Alabama shad is considered endangered by the International Union for the Conservation of Nature (*25*), and the U.S. National Marine Fisheries Service rates alewife, blueback herring (*Alosa aestivalis*), and rainbow smelt as species of concern. Among Pacific salmon species, as of 2016, the ESA listings as threatened or endangered included 11 steelhead Evolutionarily Significant Units, 9 Chinook salmon (*Oncorhynchus tshawytscha*), 3 coho salmon, and 2 each of chum (*Oncorhynchus keta*) and sockeye salmon (*Oncorhynchus nerka*) (*26*).

Declines in abundance of North American diadromous fishes are closely associated with human activities, especially those affecting the more vulnerable riverine portions of their life cycles. Humans have influenced them in many ways. However, for conservation efforts to succeed, restoration options must be available. That is, the identification and characterization of drivers of decline must be accompanied by realistic ways to reverse them. This can be seen acutely when examining North American diadromous fishes and poses the question: What remedies exist today?

Here, we briefly review the current status of North American diadromous fishes of the Atlantic (including the Gulf of Mexico) and Pacific coasts through the lens of potential remediation, referring to particular anthropogenic influences as “drivers” (Table 1). Tractability (i.e., potential remediation) was defined as the ease, or conversely the difficulty, with which a problem can be redressed, and it has two central components: (i) the availability of means (i.e., required, operational policy instruments, technologies, or institutional structures) and (ii) the acceptability of means based on social and economic considerations (*27*). In conducting this analysis, we recognize the variation in life histories and conservation pressures on the different diadromous species, including anadromous and catadromous, semelparous and iteroparous, short- and long-lived, the large differences among populations within species in life history and among rivers in human activities, and in land forms between the east and west coasts and in the duration of their Euro-American influence (ca. 400 years versus 200 years). However, we seek to identify and characterize broad trends across these species and regions.

**Table 1.** Drivers of diadromous fish decline by geographic area, major taxa of concern, and potential for remediation.

**Driver**	**Geographic area**	**Representative taxa of concern**	**Status**
Overfishing	U.S. coastal waters and coastal rivers	Striped bass, Pacific salmonids	Broadly regional, somewhat remediated
Pollution	Urban and industrialized rivers and ports	American shad, Atlantic sturgeon	Localized to watersheds, often remediated, but with exceptions including legacy pollutants
Non-native species	U.S. coastal rivers	Alosines, salmonids	Broadly regional and largely irreversible
Climate change	U.S. coastal waters and coastal rivers	All taxa	Reversible in theory but not short-term
Habitat degradation	U.S. coastal waters and coastal rivers	All taxa	Highly localized and varyingly reversible
Agricultural water withdrawals	California; eastern Oregon and Washington	Delta smelt, Pacific salmonids	Regional, dependent on climate, precipitation, water rights
Hatcheries	Northeast and Northwest United States	Salmonids	Localized and species specific, easily eliminated except in cases of proven value
Aquaculture	Northeast and Northwest United States	Salmonids	Localized to regional, due to escapees, species specific
Mortality from hydro-electric facilities	Atlantic, Gulf of Mexico, and Pacific coast rivers	Primarily American eel	Highly localized but widespread; little remediation occurring
Reduced connectivity from damming	U.S. coastal rivers	All taxa	Highly localized and available for remediation

## DRIVERS OF DECLINES OF DIADROMOUS FISHES

Through the mid-1900s, the primary drivers of diadromous fish declines were degradation of habitat (e.g., by forestry and agriculture), dams and other losses of connectivity, overfishing, and water pollution. Later or less generically relevant drivers include the effects of water withdrawals, electric generating stations, invasive species, the ecological and genetic effects of hatchery stocking (typically to mitigate losses), and indirect effects of aquaculture. Last, effects of climate change on river flow and temperature regimes are increasingly evident and overarching, and they interact with the other human activities in complex ways.

### Overfishing

Because of their economic value, most diadromous fishes have long histories of exploitation. Since European colonization of North America, many diadromous fish have been overfished, as they were in Europe (*2*, *28*). These fisheries operated both in rivers and marine waters, posing different contingencies in each habitat. With the exception of sea lamprey (*Petromyzon marinus*) (*29*) and Pacific lamprey, anadromous fishes characteristically home to their natal rivers for reproduction, resulting in genetically distinct populations (*26*, *30*, *31*). Each self-recruiting population must be managed for sustainable fisheries separately from the others, especially when there are large discrepancies in abundance and productivity among populations (*32*, *33*). These basic principles of fisheries science were recognized many decades ago (*34*, *35*), but their application can be difficult. Many anadromous species are fished in marine waters where populations mix, and management for the whole tends to result in the unintentional loss of small and less productive populations. This has been a serious problem because fisheries often catch salmon from both their own and other nations. The interceptions of North American salmon by Asian countries were progressively reduced and then eliminated, but the United States and Canada continue to intercept each other’s populations. Beyond these cases of mutual interceptions, the problem is even more acute if fishing occurs in nations (and U.S. states) that do not even produce the species in question, as there is no incentive to bargain with states producing it (*36*).

In response to the problem of interceptions, the Atlantic States Marine Fisheries Commission (ASMFC) was formed as an interstate compact, ratified by member states, and approved by the U.S. Congress in 1942. It acknowledged the need for states to jointly manage their shared migratory fisheries and affirmed their commitment to cooperative stewardship in promoting and protecting Atlantic coastal fishery resources. This was followed in 1947 by the similarly charged Pacific States Marine Fisheries Commission and, in 1949, by the Gulf States Marine Fisheries Commission. All three entities hold regular meetings with state-level representatives to share and review fishery and fishery-independent abundance data, recruitment indices, and other metrics of stock status while also considering commensurate adjustments to regulations.

A notable success for the ASMFC and other regulators such as the U.S. National Marine Fisheries Service was the recovery of the mid-Atlantic population of striped bass, which includes those populations spawning in the Hudson, Delaware, and Roanoke rivers, but most importantly, rivers flowing into Chesapeake Bay. Following its crash in the late 1970s (Fig. 1), mainly from overfishing, stringent regulations protected the moderately abundant 1982 year class of Chesapeake Bay striped bass (*10*). This cohort later spawned additional cohorts that also benefitted from the restrictions on fishing, leading to full restoration. The ASMFC has also had success restoring particular populations of Atlantic sturgeon, American shad, and river herring, chiefly by controlling fishing.

In the Pacific Ocean, one notable management achievement was the formation of the International Pacific Salmon Fisheries Commission (IPSFC) for management of Fraser River salmon and, particularly, sockeye salmon. The river basin is entirely in Canada, but U.S. interest arose from the fact that a large and variable fraction of the salmon migrate through U.S. waters as they return to spawn and are intercepted by U.S. fisheries. Rock slides in the river in the early 20th century greatly reduced the salmon runs, but in the mid-20th century, fishways at the obstruction were built, the two nations worked together, and the runs were restored to their mutual benefit (*37*). The IPSFC was dissolved in 1985 and replaced by the Pacific Salmon Commission (PSC) as part of the Pacific Salmon Treaty between the United States and Canada. The PSC manages fisheries in the transboundary rivers and coastal areas that inevitably intercept fish that originated in the other nation. For the high seas, the main mission of the International North Pacific Fisheries Commission (INPFC) (established in 1953) was resolving the problem of interceptions of North American (United States, Canada, and, especially, western Alaska) salmon in the open waters of the North Pacific Ocean by Japanese fisheries. The Union of Soviet Socialist Republics was not part of the INPFC, but now, the North Pacific Anadromous Fish Commission (established in 1992, with the INPFC dissolved in 1993) includes Russia and the Republic of Korea.

### Pollution

Two forms of pollution directly affect diadromous fishes and their ecosystems: sewage or other kinds of nutrient enrichments that stimulate algal blooms that decompose and can lead to hypoxia and toxic chemicals affecting fish health. Low dissolved oxygen caused by nutrient overenrichment often varies seasonally; algal abundance is positively related to increasing sunlight, thereby leading to hypoxia during warmer months. Hypoxia can be lethal if the fish cannot escape, and when they can escape, the reduced oxygen levels compress their tolerable habitat and may block seasonal movements. The severity and frequency of these kinds of effects varies with patterns of human density and land use from one area to another. Consequently, some regions are not afflicted with this problem, whereas, in others, it is acute. The best-characterized case on the Atlantic Coast is the Delaware River, where a zone of anoxia in late spring and early summer long blocked upriver migration of anadromous species, particularly American shad, in an area downstream of Philadelphia, PA (*38*). By the 1950s, this urban estuary was one of most polluted rivers in the world, with oxygen levels as low as zero during the summer (*39*).

Chemical contamination may cause direct mortality of diadromous fishes, such as the Exxon Valdez oil spill in Prince William Sound, Alaska in 1989 (*40*), but its influence is primarily chronic or episodic uptake with sublethal effects. For example, the effects of contaminants and of acid rain were analyzed as a possible contributor to the collapse of Chesapeake Bay striped bass (*10*). In situ and on-site bioassays revealed intermittent toxicity of environmental conditions to striped bass larvae (*41*). Studies indicated that there were water quality problems in some spawning areas (e.g., low pH and concentrations of heavy metals) with the potential to depress larval survival. However, the effects of overfishing overrode the effects of contaminants.

Because elevated levels of chemical contamination usually occur via point sources, they exhibit high geographic specificity in their effects on the freshwater phases of diadromous fishes. This was exemplified by highly contaminated adult American eels migrating from the Great Lakes watershed and captured in 1990 at Kamouraska on the St. Lawrence River. Many displayed deformities and lesions, and their levels of polychlorinated biphenyls (PCBs), Mirex, and other pesticides were 10 to 100 times higher than in the migratory adult American eels from an uncontaminated tributary (*42*). However, contamination may be experienced broadly, such as acid rain. Low pH in streams can cause mortality and impair smolt development in juvenile Atlantic salmon, thus episodic acidification is affecting its conservation and recovery in the northeastern United States (*43*).

Pacific salmon may spend extensive or developmentally critical periods in freshwater environments that expose them to contaminants. In general, the upper parts of watersheds are clean, and juveniles become exposed when they feed in and migrate through the lower reaches that are more typically in farmland, suburban, and urban development and in estuaries that may be highly developed into cities and ports (*44*). The migratory life cycle makes it difficult to identify the role of contaminants in population viability for three reasons. First, phases in the lives of salmon are spent in regions with different types and levels of contaminants as they move from agricultural to industrial waterways, for example. Second, the movements may be in a single life history loop (i.e., out and back, followed by reproduction and then death in the semelparous species) or back and forth repeatedly in the iteroparous species, including skipped migration and various alternative patterns affecting contaminant exposure. Last, it is difficult to determine the effects of contaminant exposure on a background of natural mortality and other human influences. The rivers and estuaries where chemical contaminants are most concentrated are often also affected by other forms of human development affecting migratory fish populations, such as the Columbia River (*45*) and San Francisco Bay (*46*), making it difficult to ascribe losses to one cause or another. These contaminants are seldom found at lethal levels, but they can hinder the functioning of the immune system and increase the susceptibility of juvenile salmon to pathogens (*47*). However, an exception to the pattern of primarily sublethal effects of contaminants is the rapid mortality of adult coho salmon after exposure to certain chemicals found in urban streams. Up to 90% of the females may die in their natal stream before spawning (*48*, *49*). Recently, it was determined that this acute mortality results from chemicals derived from an antioxidant in automobile tires (*50*).

### Agricultural water withdrawals

As with contaminants, water withdrawals for agriculture and other uses are patchy on the landscape and thus affect some populations but not others. Coastal watersheds from northern California to southeast Alaska tend to be small and well watered—especially from late fall through early spring—and many are steep. Consequently, logging is often the primary land use or agriculture that does not require extensive irrigation. However, land on the east side of the coastal mountain ranges is much drier overall, and the seasonal precipitation patterns are different. In parts of the Klamath (*51*) and Columbia River (*52*) basins, water diversions can be very important. Storage and release of water from dams may cause marked reductions in flow during parts of the year and higher than natural flows during other periods, as needed to meet the demands of agriculture and electric power generation. The adaptations of fishes and other organisms to the seasonal patterns and levels of variability of flow regimes are essential to their persistence (*53*). As with other stressors, of course, there are complex interactions between, for example, flow, temperature, contaminants, and physical habitat alternation (*54*). The once-abundant and diverse salmon runs of the Sacramento–San Joaquin River system have suffered from multiple alterations in the watershed and estuary (*21*, *55*). Similarly, California’s delta smelt (*Hypomesus transpacificus*) is in jeopardy from a complex of processes including changes in the estuary’s habitat and salinity regime, among other factors related to water management (*56*).

### Fossil fuel and nuclear electric generating stations

The combined effects of electric generating stations on anadromous fishes have been felt on a number of rivers. Facilities that withdraw water from rivers may kill large numbers of fish eggs and larvae through entrainment and by impinging larger individuals against intake screens; plants may also alter local temperature regimes by discharging heated water (*57*). These impairments have been most pronounced in the Hudson River Estuary, where more than 10 fossil fuel stations use once-through cooling. It is estimated that entrainment for five large mainstem power plants on the Hudson River induced conditional mortalities of striped bass of 11 to 22%, of American shad of 14 to 21%, and of river herring of 4 to 11% (*58*); impingement added additional mortalities (*59*). Power plants affect other Atlantic rivers too, e.g., on the Delaware River, the Salem and Hope Creek nuclear power plants pump over 3 billion gallons of cooling water once-through per day from the estuary (*60*), entraining and impinging diadromous fishes. Likewise, on Chesapeake Bay, 13 power plants withdraw and discharge 8 billion gallons per day (*61*). However, owing to a greater regional reliance on hydroelectric power, rivers of the west coast are less affected by these types of facilities and operations.

### Hatchery supplementation

Fish culture has an ancient history but the first experiments on artificial propagation of Atlantic salmon took place in Europe in the 18th century. By the 19th century, hatchery programs on North America’s east and west coasts were developed to offset what were recognized as excessive fishing rates because limiting the catch was unpalatable (*62*, *63*). Hatchery propagation is attractive because, as long as the fish are under human control during the embryonic and juvenile stages, their survival rates far exceed those of fish in rivers, lakes, and at seas. However, the high survival rates often result in excessive fishing on wild fish that mingle with their hatchery counterparts, and competition, predation, diseases, and other ecological processes intervene as well. Thus, in some cases, the total number of fish increases, but, in other cases, it is much less clear, and some substitution of hatchery for wild individuals may occur (*64*). Consequently, the role of hatcheries in restoring anadromous fish on the Atlantic, Pacific, and Gulf coasts is highly controversial and complex, and they have often failed to halt declines and restore former levels of abundance (*63*).

The steep decline of the Chesapeake Bay striped bass in the late 1970s led to a program to stock hatchery-reared individuals in the Bay, with 7.5 million juveniles released from 17 hatcheries between 1985 and 1993. Analyses concluded that stocking of striped bass may have enhanced recovery in localized areas of Chesapeake Bay but that the contributions to population recovery were far greater from reducing fishing mortality than from stocking (*10*). Harmful hatchery-based restoration of striped bass occurred in the 1960s and 1970s on the Gulf Coast where individuals were stocked that had been spawned from more readily available Atlantic Coast populations. It was later determined that these individuals were less fit for Gulf watersheds than was the native population. Nonetheless, introgression occurred and a long-term program to identify and breed individuals with native genetic signatures for subsequent stocking was needed (*65*).

**Fig. 2.**
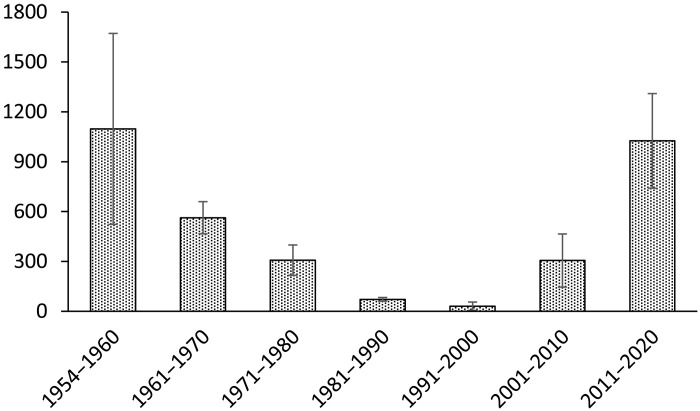
Average counts (±SE) of mature, returning sockeye salmon, *O. nerka*, at a weir at Redfish Lake Creek in Idaho, the population’s primary spawning site, from 1954 to 1964 (*184*), and at Ice Harbor Dam, farther downriver on the Snake River from 1962 to 2020 (values averaged for the overlapping years). The population’s steep, multidecadal decline resulted in its listing as endangered under the U.S. ESA. It was saved from extinction and has partially recovered, but only with continuing artificial propagation (*185*). Ice Harbor Dam data were collected by the U.S. Army Corps of Engineers, obtained from the Data Access in Real Time (DART) website: Columbia River DART, Columbia Basin Research, University of Washington (2021). Adult Passage Annual Counts. Available from www.cbr.washington.edu/dart/query/adult_annual_sum.

Salmonids have been the focus of far more hatchery stocking than other U.S. anadromous fishes. Naish *et al.* (*66*) reported in 2007 that, over the prior 10 years, annual releases of early life stages in New England of hatchery-produced Atlantic salmon averaged 10 million (and far more on the Pacific Coast). Nonetheless, Atlantic salmon in U.S. waters remain near record low levels; just 869 adults returned in 2018 (U.S. Atlantic Salmon Assessment Committee 2019), and, in most U.S. rivers where they persist, they are in jeopardy. Experience with Pacific salmon is, as with other factors, mixed and complex. In parts of the Columbia River basin, for example, hatcheries were constructed to mitigate for runs that were eliminated by impassable dams; without the hatchery, the upper reaches would not produce any anadromous fishes. However, in many other areas, there are controversies about the extent to which hatchery production has resulted in fishing pressure that is unsustainable by comingled wild populations, overtaxed the carrying capacity of ecosystems, and altered the genetic makeup of the populations under artificial production—see also reviews generally supporting hatchery production (*67*, *68*). Regardless of how they are perceived, hatchery programs are now so extensive (*69*, *70*) that they must be viewed as a large part of the overall ecology, management, and evolution of salmonids (e.g., Fig. 2).

### Effects of aquaculture

Aquaculture is distinct from hatchery production in that cultured fish are not purposely released to feed in common with other fishes but are confined to net pens, tanks, or other containers. Thus, they are more analogous to feed lots as distinguished from free-ranging livestock. As reported by the Food and Agriculture Organization (FAO), salmonid (Atlantic, coho, and Chinook salmon, and rainbow trout *O. mykiss*) aquaculture was less than 200,000 metric tons annually up to 1980 but then increased rapidly and exceeded 2,000,000 metric tons by the beginning of the 21st century. Aquaculture now produces more than half the salmon consumed on a global basis based on FAO statistics, thus exceeding wild salmon production. Some of this production is not only within the native range of salmon species, such as Atlantic salmon net pens in Norway, but it also involves species outside their native range in the Northern Hemisphere such as Atlantic salmon in British Columbia and Washington and to areas where salmonids are not native, such as New Zealand and Chile.

Some of the concerns about aquaculture stem from the tens of millions of farmed salmon that have escaped into the wild. Atlantic salmon raised on farms now far outnumber wild Atlantic salmon returning to rivers, and escapes occur in all aquaculture regions both through regular, low-level “leakage” and through much larger episodic events from storms and poor maintenance. In their native range, an estimated 2 million farmed salmon escape each year into the North Atlantic (*71*).

There are risks to these escapes. Vagrant farmed salmon can interbreed with wild individuals, and the progeny have lower fitness for life in the wild compared to native fish (*71*). Hybrids may occur and thus lower the overall reproductive success of the blended population. Even if the escapees do not reproduce, they may occupy space and thus compete with wild individuals. Moreover, high densities of farmed salmon in pens can support large numbers of sea lice (*Lepeophtheirus salmonis*) and other copepods that parasitize juvenile salmon that swim in the vicinity of the net pens. The ecology of these parasites is complex, and they occur at low levels naturally, but there is increasing evidence that expanded sea lice populations associated with salmon net pens constitute a threat to wild salmon in both the Atlantic (*72*) and Pacific oceans (*73*).

There is also concern that some of the many pen-reared Atlantic salmon that escape each year might survive and interbreed or compete with native Pacific salmon. The first catches of Atlantic salmon in North Pacific waters occurred in 1987, and between 1988 and 1995, 97,779 escapes were reported, with 9096 caught in marine waters between Washington and Alaska and another 188 in fresh waters (*74*). However, no populations have been established and, to date, they are not widely deemed as great a concern as are other stressors.

### Non-native fish species

In Atlantic rivers, the low predation regime on anadromous fishes hypothesized to have prevailed under natural conditions centuries ago has been altered by the addition of non-native predators (*1*). In the Northeast, non-native black bass (*Micropterus* spp.) are ubiquitous piscivores in rivers that can be especially destructive to juvenile and adult American shad, blueback herring, and alewife (collectively known as alosines), as can predatory blue catfish (*Ictalurus furcatus*) and flathead catfish (*Pylodictus olivaris*) (*75*–*77*). These predators are native to North America, but the non-native piscivore, northern snakehead (*Channa argus*), is now established in Chesapeake Bay (*78*). Common carp (*Cyprinius carpio*), introduced to American waters from Eurasia in the 1800s, may comprise a substantial portion of fish biomass in rivers all over the United States and consume the eggs of anadromous fishes (*79*).

In the Hudson River alone, more than two-dozen non-native fish species can prey upon various life stages of its anadromous fishes (*80*). Moreover, in the Hudson and elsewhere, non-native organisms other than fishes may also affect them. Strayer *et al.* (*81*) showed that the ecological changes caused by the colonization of the Hudson River by the Eurasian zebra mussel (*Dreissena polymorpha*) lowered abundance and growth and shifted the distributions downriver toward tidewater for early life stages of striped bass and American shad.

In the Pacific Ocean basin, the diversity and effects of non-native fishes are generally greatest in the southern part of the range, as with so many other effects, and diminish northward (although with some exceptions). Not all non-native fishes have significant ecological interactions with native anadromous species, but many are predators, including striped bass and largemouth bass (*Micropterus salmoides*) in the Sacramento–San Joaquin river system (*82*), where their effects are exacerbated by artificial light at night (*83*). The Columbia River has substantial and growing populations of smallmouth bass (*Micropterus dolomieu*) (*84*) and walleye (*Sander vitreus*) as well as native predators (*85*, *86*). These predators are likely to benefit from the warming conditions projected to occur in the future (*84*, *87*).

In general, the threats are from predatory non-native fishes transported across long distances (e.g., East to West Coast), but in some cases, they come from smaller shifts in distribution. For example, northern pike (*Esox lucius*) are native to some parts of Alaska but typically do not exert an ecologically dominant effect on salmon. However, anglers have transferred them to regional basins where they are not native, and they have had strong negative effects (*88*, *89*).

### Climate

Changing climate is altering the distributions of diadromous fishes. For example, in earlier centuries, the boreal rainbow smelt ran in rivers as far south as Virginia, in the Delaware until the 1880s, but vanished from the next major northern river, the Hudson in the 1990s, and its range and numbers of rivers inhabited are continuing to shrink northward (*90*). Alewife have become absent from rivers at the southern end of their range in South Carolina and, possibly, in North Carolina (*91*). Moreover, climate change may reduce the total suitable habitat in the sea, altering the marine distribution of river herring (*92*). However, striped bass were caught in Newfoundland and Labrador, farther north than they had been seen before (*93*). Similarly, there are increasing reports of Pacific salmon in Arctic rivers, where they had previously been absent or scarce (*94*, *95*).

In addition to shifts in range, climate change affects organisms in many other ways, especially from increases in temperature (higher summer peaks and earlier warming in the spring and later cooling in the fall) and changes in river flow regimes. Water temperatures track increases in local air temperature in many cases, but this can depend to some extent on the sources of the water. For example, typical runoff streams will track air temperature closely, whereas those fed by groundwater will track it more slowly, following mean annual air temperature more than the short-term fluctuations. Last, streams fed by glaciers and snowfields may remain cold as long as there is snow and ice left to melt, so the diversity of water sources within a basin is important (*96*, *97*).

River flow regimes can change with the climate in direct and indirect ways. If there are changes in the magnitude or timing of precipitation, then river flows will be altered. However, the more common effect, especially in higher elevations and latitudes, is the shift from snow-dominated to rain-dominated flow regimes and the reduction in winter snowpack (*98*). The reduction in summer flows that may result from diminished snowpack, combined with higher summer temperatures, may be especially detrimental to cold-water fishes (*99*).

Climate change is also evident in changes in phenology—the seasonal timing of life history events such as migration, breeding, and hatching of juveniles. Monitoring in the Penobscot River in Maine revealed that the median capture date for Atlantic salmon in the Penobscot River advanced by 1.3 days per year between 1986 and 2001 and by 1.2 days per year between 1983 and 2001 for alewife in the Androscoggin River (*100*). Likewise, on average, the initiation of seaward migration of Atlantic salmon smolts has occurred 2.5 days earlier per decade throughout the North Atlantic basin (*101*).

In general, alosines are much more responsive to changes in temperature than are salmonids (*102*). The relationship between temperature and American shad migration timing was known nearly a century ago (*103*), and this has been borne out by many subsequent studies on alosines in their native Atlantic range (*104*, *105*) and after transplantation to the Pacific Coast (*102*). Expectedly, many rivers are becoming warmer earlier in the year, and migration earlier in the spring is the result. This creates a possible mismatch if the fish are cued to migrate and breed at a time that is maladaptive, given subsequent conditions affecting them or their offspring.

A unique climate challenge will occur for anadromous fishes in the Gulf of Mexico. Gulf sturgeon (*Acipenser oxyrinchus desotoi*) and striped bass depend on limited numbers of cool spring-fed refuges to survive seasonally high temperatures in Gulf rivers (*106*, *107*); warming may impose even greater stresses on these populations. Moreover, because of their east-west distributions, Gulf populations of these species have no prospects for latitudinal adjustments to climate, thereby threatening them with extinction.

Last, the interaction between dams (see also below) and climate change exemplifies the complexity of factors affecting migratory fishes. In addition to the obvious effects on flow regimes, which were often directly linked to the dam’s purpose, dams also commonly affect downstream thermal regimes. Water released from below dams is often cooler in the summer compared to ambient conditions (*108*), and this may mitigate some climate effects (*109*), but dams also may elevate winter temperatures, with complex ecological and evolutionary effects (*110*).

### Habitat degradation

Habitat destruction and degradation are widespread drivers of diadromous fish population declines. Habitat destruction, i.e., the direct landfilling, removal, or other severe disturbance of habitat, can eradicate or diminish spawning grounds, nurseries, and feeding areas. For instance, in the Hudson River Estuary, one-third of lower Manhattan is made up of landfill, and this fish habitat is irretrievably lost. Similarly, many of the habitat changes in San Francisco Bay, the estuary of the Sacramento–San Joaquin River system (*55*), are unlikely to be remediated. Estuaries and coastal areas around the world have been greatly modified for centuries (*111*). Varying degrees of habitat degradation are prevalent in many if not most river systems, with ports and urban centers, suburban expansion, agriculture, grazing, and forestry activities in many basins.

Expectedly, the importance of habitat drivers varies among regions. For example, although no longer a major factor on the Atlantic Coast, logging was identified as the fifth most important among 15 drivers affecting California salmonids, including anadromous forms (*24*). Many habitats used by diadromous fishes have been adversely affected, including recent activities and systemic legacy effects on many northeastern rivers caused by Colonial mills on sediment transport (*112*). The net result has been long-term alteration from wide and shallow river beds with braided channels, coarse sediments, and woody debris, to straighter and deeper U-shaped channels lined with fine sediments that had been trapped by the dams. It remains unknown how these changes affect diadromous fishes, but it is clear that they evolved to rely upon rivers of a certain long-term morphology that has been superseded by a novel type. There are, of course, extensive efforts to restore rivers used by these and other fish communities (*113*, *114*). In many cases, these efforts are successful, especially in areas where forestry, grazing, and agriculture are the dominant land uses, but human expansion presents challenges that make some thoughtful scientists pessimistic about the prospects (*115*). Thus, in many ways, while stream restoration and reconnection will improve habitat in many areas, cities will affect salmon with only modest scope for remediation.

### Damming and reduced connectivity

There are more than 80,000 dams 6 feet high or more in the United States and 2.5 million smaller ones (*116*, *117*). Many dams have fish ladders or fish elevators to pass fish over them but that perform poorly, and individuals that succeed in passing them may have additional dams to surmount before reaching their spawning grounds, causing progressive reductions in their numbers and cumulative delays in their attempts to reach spawning locations. To surmount some dams, either in upstream or downstream directions, managers may use “trap and haul” in which fish are collected and trucked to their destinations (*118*). This approach is frequently used as a short-term form of necessity but has been employed sustainably over time. Fish passage facilities also are typically designed to favor certain species, and aspects of their design may make them less effective for sturgeon (*119*), lamprey (*120*), or shad (*121*). Hydro-dams also sometimes cause direct mortality of migrating diadromous fishes. Female American eels traveling from the Great Lakes watershed toward the sea through the St. Lawrence River have an estimated survival probability as low as 1.4% due to injury by the blades from hydro-dam turbines (*122*).

More broadly, dams shrink or alter accessible habitat and, consequently, spur declines in diadromous fish production. Overall, fish access to more than 70,407 km of rivers to the next upstream barriers on them is impeded by terminal dams across the Gulf of Mexico and Atlantic and Pacific coasts (*123*). U.S. rivers are among the least connected for diadromous fishes in the world (*124*). Moreover, numerous first dams above tidewater lack engineered passage facilities for diadromous fishes. For American shad, approximately 4000 of an original 11,200 km of spawning habitat have been lost to dams (*15*). Historic alewife populations in Maine for the years 1600 to 1900 were assessed using 19th and 20th century catch records and data on dams dating to the 1600s (*125*). Obstructed spawning access in nine watersheds reduced the annual alewife productivity per watershed to 0 to 16% of virgin estimates, equaling a cumulative lost fisheries production of 11 billion fish from 1750 to 1900.

Impassable dams have had a fundamental effect on Pacific salmon and other diadromous fishes in the U.S. Pacific Northwest and, to a much lesser extent, in Canada and Alaska. Dams on many of the large rivers and especially the Sacramento–San Joaquin, Klamath, and Columbia River systems extirpated many populations of these fishes, and these habitats remain inaccessible. Many smaller rivers, both tributaries of these large rivers and independent rivers along the coast, were dammed without passage facilities, and the dams contributed greatly to the numbers of populations that went extinct or are in jeopardy (*18*).

The nature of many west coast rivers makes them exceptionally well suited to hydroelectric power, and their production of power from dams alters rivers in many ways that affect salmon and other native migratory fishes, notably white sturgeon (*Acipenser transmontanus*) and Pacific lamprey. These many effects are detailed and discussed in National Research Council reports (*52*, *126*), including the elimination of habitat if the dams were built without provision for fish passage, and the alteration of natural flow regimes, gas super-saturation as water spills over the tops of dams, direct injury and death during migration through turbine and bypass facilities, transition of riverine to lacustrine (reservoir) habitats with attendant changes in the native fish community [e.g., more conducive to native predators such as northern pikeminnow (*Ptychocheilus oregonensis*), and predatory non-native fishes such as walleye and smallmouth bass], and delays in migration downstream and upstream.

## POTENTIAL FOR REMEDIATION

The litany of drivers outlined above falls into several categories in terms of potential remediation. If a species is affected by only one, then the prospects for recovery are much better than they are for species affected by several drivers, especially if some are largely intractable. In a review of the status of Atlantic diadromous fishes, among North American species and populations, only coastal migratory striped bass had recovered, and this was due to a strong management response to the single primary driver of overfishing (*16*). In contrast, Atlantic salmon populations and some Pacific salmon populations in the United States face the simultaneous problems of damming, climate change, non-native species, urbanization, water withdrawals, and habitat alteration from diverse land-use practices, all complicated by hatchery production and ongoing fishing pressure. We consider the drivers below in relation to their potential remediation, recognizing that there is really a continuum of conditions rather than discrete categories and that the extent to which drivers have been addressed varies among geographic regions.

### Drivers already largely remediated

Overfishing, long a primary driver of diadromous fish declines, has diminished substantially due to the combined efforts of the Atlantic, Gulf, and Pacific States fisheries commissions that began in the 1940s. Managing fisheries is not an exact science, and some overfishing of diadromous species still occurs. However, the system of three compacts of coastal states operates with checks and balances among their constituents and among fishery stakeholders within them, in addition to conducting ongoing monitoring that allows for frequent adjustments to landings, with the net result being a considerable lessening of overfishing as a contemporary driver. In the open ocean, Atlantic salmon from a wide range of origins in North America and Europe feed in international waters and especially on the west coast of Greenland and the Faroe Islands, where fisheries exploited them heavily (*127*). Thanks, in part, to international groups such as the North Atlantic Salmon Conservation Organization and agreements among nations, these interceptions have been greatly reduced, but fishing including significant illegal, unreported, and unregulated may still occur (*36*).

Among Pacific salmon, interceptions on the high seas are a thing of the past, due to the successes of the INPFC. However, the geo-political boundaries result in three coastal states (California, Oregon, and Washington, going northward), then Canada, and then the state of Alaska. The nature of salmon migrations is typically (but not exclusively) northward from their home rivers when they enter the ocean and, thus, southward on their way back. Consequently, Alaskan fisheries are much more likely to intercept salmon produced in Canada and the other states than the other way around. Broadly speaking, salmon fishing is well regulated in terms of maintaining overall yield, but this can result in overfishing some smaller or less productive populations, especially wild populations fished in common with hatchery populations (*128*). For large population complexes such as Bristol Bay, Alaska, managers of sockeye salmon use several sources of information to manage for scientific-based escapement goals and detailed in-season assessment (*129*, *130*). However, myriad small populations have minimal assessment and may chronically fail to meet escapement goals (*131*). This conflict in managing for biomass versus biodiversity is at the heart of management for species such as anadromous fishes whose migrations cause genetically discrete populations to be exploited in common. Nevertheless, humans have a strong measure of rapid control over fishing, although our willingness to apply that control varies with socio-political pressures.

### Continuing to be remediated

Water pollution is certainly still a human and ecological health issue, but many forms of it have been substantially reduced as a direct driver of diadromous fish declines. Although pollution in less environmentally conscious times was not severe enough to be a major driver in most rivers, some waterways experienced profound chemical and sewage contamination. However, in U.S. rivers today, pollution is not typically viewed as the main cause of current diadromous fish declines, a circumstance largely attributable to the benefits provided by the Clean Water Act of 1972 and subsequent actions. Acid rain, once a prominent environmental threat in North America and Europe, was reduced markedly (*132*). However, some contaminants, notably harmful organic compounds such as PCBs and DDT are legacies of past use, and they cycle within the environment and degrade very slowly. In these cases, reduction in exposure is a long-term process, and current levels may still cause harm (*45*). Moreover, previously unidentified, highly diverse “contaminants of emerging concern” are being used (*133*, *134*). Consequently, the assessment of contaminant effects will continue to be important and, in some river systems, will affect fish survival in direct and indirect ways. Given the tendency of chemicals to be deposited in estuaries, diadromous fishes are more vulnerable to these kinds of factors than are fishes living exclusively in marine or freshwater habitats.

Open-cycle electric generating stations have caused substantial mortalities, mostly of early life stages, for anadromous fishes in many rivers, mainly through entrainment and impingement. There are conflicting viewpoints on the reductive effects of power plants on fish populations. One analysis estimated that the combined effects of the power plants on the Hudson River reduced the cohort sizes of striped bass by about 20% (*135*). However, a review of 40 years of research concluded that reducing entrainment and impingement mortality via regulation of cooling water intakes will not measurably improve recreational or commercial fish populations (*136*). Power plants continue to harm diadromous fishes, but, as the plants age and undergo attrition, it is expected that many will be replaced by units with closed-cycle cooling or by alternative energy sources, eventually reducing their effects. The nearly 1040 MW nuclear Indian Point Energy Center on the Hudson River was decommissioned in 2021.

### Drivers that are typically irreversible

Decades of experience has shown that it is difficult or impossible to extirpate a non-native species once it finds suitable biotic and abiotic conditions and becomes established, and this is certainly true for fishes (*137*). For example, the Hudson and most rivers in the United States now have several or many non-native fishes and other taxa that prey on and compete with native diadromous fishes. When northern snakehead was discovered in a tributary to the Hudson River in 2009, intensive control efforts were initiated. This threat appears to have been eradicated but the species remains established elsewhere in the United States, and so, future spread is always possible, and such success stories are more the exception than the rule. In some cases, the non-native species was deliberately introduced typically but not invariably for recreational fishing. These commonly include large, predatory fishes such as northern pike, largemouth and smallmouth bass, striped bass, and walleye. Local anglers often lobby agencies to manage these predators for sustainable fisheries rather than seeking their elimination or reduction, as they might if protection of juvenile salmon was the goal.

### Drivers that are reversible in theory but not in practice

Because diadromous fishes rely on both marine and freshwater habitats, and corridors between them, the effects of climate change on these fishes are especially numerous and complex. Deleterious effects include temperatures that favor native and non-native warm-water fishes that prey on cold-water juveniles in fresh water (*87*); mortality of mature adults migrating upriver or on the spawning grounds (*138*–*140*); increased incidence of unfavorable physical ocean conditions, such as “marine heat waves” (*141*, *142*); and complex ecological effects at sea. Shifts in precipitation—source (rain or snow), magnitude, and timing (*143*, *144*)—are especially important for juveniles of the stream-rearing species, and also species that hold in rivers as adults during the heat of the summer, when flows may be limiting, before spawning.

The effects of climate change can, in theory, be ameliorated by major reductions in the release of anthropogenic greenhouse gases, which would translate to cooler water temperatures. However, a modest reduction would not stem the momentum of rising temperatures entrenched in current planetary processes. At this time, there is little evidence that sufficiently rapid and meaningful societal alterations will be achieved to begin to reverse climate change, and the effects are likely to increase (*145*). Thus, at least in the near term, we regard these effects as not available for remediation, at least at the scales needed for diadromous fish conservation.

Drivers available for remediation: The harmful effects of at-sea aquaculture can be reduced through better containment of the farmed stock. In addition, it may be that gene editing could produce strains of fish that are truly sterile and maybe even non-migratory should escapes occur. However, regulating aquaculture primarily affects only salmonids and, in localized regions, and thus will not have broadly regional results. Hatchery augmentation affects a much wider geographic range and most salmonid species. Artificial propagation needs to be carefully considered in light of the reproductive potential of co-occurring wild fish and the harm that could be done to them by supplemental stocking, with consideration of fishery management, ecology, and genetics. Such assessments are becoming part of the management process in some areas (*146*).

Habitat degradation is a broad driver of decline that can be reversed in some cases. Society has made claims to river banks and channels for cities, factories, highways, ports, and other hardened infrastructure that would largely preclude remediation. However, substantial investments in successful restoration have been made in many areas. For example, dikes were removed from what had been agricultural land, allowing the naturalization of the Nisqually River estuary in Puget Sound. At 364 ha, this is the largest tidal marsh restoration project in the northwestern contiguous United States (*147*). Similarly, removal of dikes in the Salmon River estuary, along the Pacific Ocean coast of Oregon, increased accessible habitat by 145 ha and expanded life history diversity of salmonids moving between the river and the ocean (*148*–*150*). Each site, of course, has its own sets of constraints and trajectories of recovery, and there are limits to the number of such opportunities, but it is important to take them when they present themselves.

Another readily available restoration option is to increase connectivity in watersheds by the improvement of culverts. Culverts are less obvious and receive less attention than dams, but they are much more numerous and, collectively, hinder or prevent access to large fractions of many basins such as the lower Columbia and Willamette rivers (*151*). Throughout North America, many culverts are poorly designed for fish movements, having perched outlets or shallow flows. For instance, culverts were estimated to more than double the amount of time required for river herring to traverse a Massachusetts river than in the absence of culverts (*152*). Because culverts are a feature primarily of smaller flowages, their improvements are likely to favor diadromous species such as juvenile salmonids that extend far into drainage systems, including first order streams. The great numbers of culverts in some basins mean that, together, they could pose a substantial challenge to migratory fish (*153*), and even the costly processes of permitting and repair do not ensure success (*154*).

**Fig. 3.**
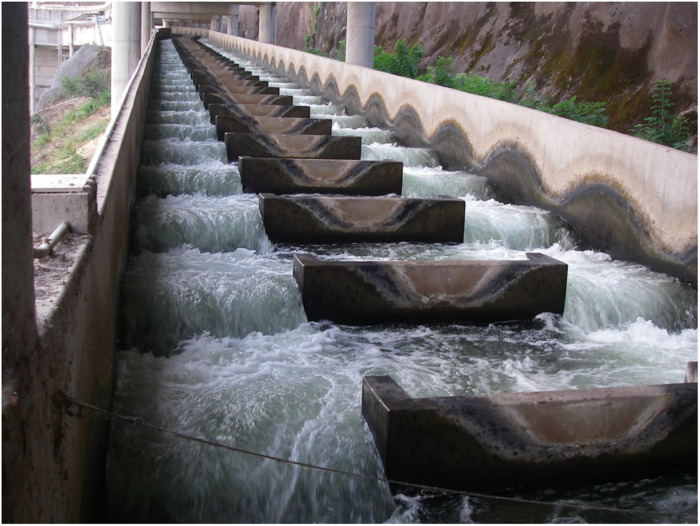
Fish ladder at the Lower Granite Dam, Snake River, Washington. Ladders such as this allow passage at dams that would otherwise extirpate upriver runs but can delay upriver migration. Photo credit: Thomas P. Quinn.

All in all, dams are the most important and most remediable driver for the restoration of American diadromous fishes. Many dams on rivers with diadromous fish populations have engineered fishways to “pass” individuals over them on the spawning runs (Fig. 3). A few work well enough to sustain moderate fish runs, although likely far below original abundances. Considerably, more common are fishways that sustain relict or small runs, like many on northeastern U.S. rivers. Reduced abundances are especially pronounced where there are two or more dams en route to spawning areas upriver.

Long-term reliance on engineered fish passage facilities in three major northeastern rivers (Merrimack, Connecticut, and Susquehanna) showed failure to achieve even modest population recoveries for American shad despite unambitious restoration targets (*155*). The Susquehanna’s runs of American shad past the fourth dam to its historical spawning grounds have fallen as much as five orders of magnitude, in some years to single digits (*16*), and fewer than 3% of the shad successfully negotiated the first three dams. Many dams also hinder or prevent passage downstream for spent adults, minimizing multiyear spawning runs by adults and sacrificing the future higher fecundity of larger individuals, and often compromise emigration of juveniles as well.

Dam removal is a proven and highly successful means of diadromous fish restoration (*156*). After centuries of building dams, more and more are being removed (*157*). The removals are motived by concerns for safety in some cases and obsolescence in others, but frequently, the reconnection of river corridors for fishes is an important, if not the prime motivation (*158*). There is clear evidence, from many rivers, that migratory fishes rapidly expand upstream to take advantage of the newly accessible habitat, including examples from both coasts, anadromous and catadromous species, and diverse taxa (*159*–*162*).

On the U.S. East Coast, Edwards Dam was constructed on Maine’s Kennebec River in 1837, where it blocked access for migratory fishes to the next 27 km of river extending to the next mainstem dam. Sited not far above tidewater, Edwards Dam immediately caused steep declines in Atlantic sturgeon, shortnose sturgeon, Atlantic salmon, striped bass, American shad, and river herring (*163*). Long-term efforts to remove Edwards Dam were controversial. It generated electricity, but only 3.5 MW, a small amount for a large dam (280 m long and 7.3 m high), and less than one-thousandth of Maine’s needs. The alternative option of fish passage specified by the U.S. Fish and Wildlife Service would have cost $9 million and helped restore only three of the river’s anadromous fish species, whereas removing the dam would have cost 40% less and would have helped restore all 10 species (*164*). In the end, the Federal Energy and Regulatory Commission concluded that the dam’s environmental costs outweighed its economic benefits.

Removal of Edwards Dam in 1999 was precedent setting as the first of an actively generating hydro-dam in the United States. Almost immediately following its demolition, anadromous species were observed in the newly accessible river reach. Most notably, reopening these waters allowed these fishes access to a tributary, the Sebasticook, where the run of alewives and blueback herring grew from zero over the 162 years before removal of Edwards Dam to nearly 6 million in 2018 (*165*). The renewed river herring run in the Sebasticook provided numerous benefits, including the focus for a community alewife festival and as a much-needed source of bait for coastal trapping American lobster (*Homarus americanus*) (*166*). However, even clear successes such as the renewal of large alewife runs in the Sebasticook are qualified in that access to substantial habitat above the current lowermost mainstem dam in the Kennebec remains blocked by ineffective passage (*167*). Removal of the remaining four lowermost dams on the Kennebec would provide a freely open pathway to another 135 km of mainstem and to the river’s major Atlantic salmon spawning tributary, the Sandy River. The electrical production of these four dams, if removed, could be replaced locally with ~204 ha of photovoltaic arrays (*168*) or regionally with the large-scale offshore windpower installations planned for coastal Maine.

The benefits of dam removal are heightened when coordinated with other dam removals and associated actions conducted as part of whole-system restorations. For example, Maine’s Penobscot River Restoration Project, completed in 2016, featured removal of the two dams closest to the sea, bypassing of a third with a nature-like fishway, and improved fish passage at four other dams, providing enhanced access to 3200 km of historical habitat while simultaneously increasing total hydropower production (*169*). The Penobscot project is expected to foster recoveries of a dozen migratory fishes; annual alewife runs grew from the hundreds and thousands before the restoration to 2.8 million by 2018.

## DISCUSSION

As we advance into the Anthropocene, human activities have extirpated many diadromous fish populations or driven them to relict levels (*16*). Very few diadromous fish populations in the United States are as large today as they were before European colonization other than those in Alaska. This massive conservation failure is especially conspicuous, given the tremendous economic, cultural, and ecological importance of these fishes. It is crucial to implement the most effective remaining options to stabilize and restore their populations. Damming of rivers is the most common and widespread significant driver of declines, and dam removals remain the most available remedy for meaningful restorations. Moreover, the benefits of dam removals can be readily assessed directly through monitoring of fish movements and abundances. Many other factors have contributed to these population reductions; some offer limited potential for remediation, some have been addressed to some extent already, and others are less tractable, or more localized in effects. However, none remain as efficacious nor as tractable as dam removals.

Focus on dam removals as the most effective and available driver also dovetails with other societal considerations of dams today. The era of dam construction in America ended some time ago and, in the Northeast, was launched in the Colonial era. Consequently, most dams in the United States, and especially in the Northeast, are aged, and many have already exceeded their planned life spans (*170*). Because of their age and disrepair, many dams in the United States are in danger of failing when challenged by the increasingly severe weather associated with climate change, and some already have done so (*170*). The regular maintenance needed to extend the longevity of dams can be more expensive than removing them (*171*). Since the 1970s, the number of dams removed for safety reasons has increased, but, as a proportion of all removals, this rationale has increasingly been replaced with environmental and economic considerations (*158*). Moreover, although hydro-dams have often been constructed on larger rivers, the vast majority of American dams were not built for electricity production. Many of these are abandoned, derelict, or offer little societal value. It is important that nongovernmental organizations, states, and the federal government continue to assess both the physical and ecological circumstances of these dams for new opportunities to begin removals.

**Fig. 4.**
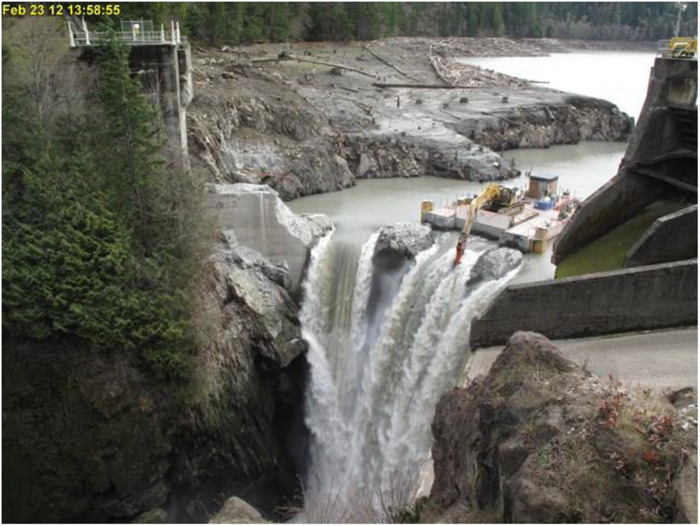
Removal, in 2014, of largest dam in the United States to date, 64-m-high Glines Canyon Dam on the Elwha River, Washington, 21.6-km river from the sea. Chinook, coho, chum (*O. keta*), pink salmon (*Oncorhynchus gorbuscha*), sockeye salmon, steelhead trout (*O. mykiss*), and Pacific lamprey have recolonized the reach upriver of the dam site (*186*). In addition to the benefit of improved upstream migration by species formerly limited to habitat below dams, dam removal can also allow the re-awakening of dormant life history variation, as landlocked populations resume anadromy (*187*, *188*), contributing to population viability (*174*). Photo credit: National Park Service.

As renewable forms of energy production have become scaled to industrial levels, the impact of dams on river flows and fish populations require critical reevaluation. In some cases, it may be possible to consider the removal of hydro-dams and replacement of the foregone energy with one or more in situ or ex situ alternative energy sources such as solar and wind power (*168*, *172*). In other instances, hydro-dams also provide essential or valued services such as irrigation, flood control, transportation, and recreation (reservoirs are also often valued for boating and other uses). Consequently, for some dams, the arguments for their continued operation based on broad societal benefits may outweigh ecological considerations. However, the four large hydro-dams on the Klamath River system, spanning northern California and southern Oregon, are now slated for removal, and thus, removals that seemed unthinkable a generation ago are taking place. This follows the West Coast precedent of the recent removals of the large Elwha and Glines Canyon dams on Washington’s Elwha River (Table 2 and Fig. 4).

**Table 2.** Selection of representative dams removed in the United States at least, in part, for the benefit of diadromous fishes (along Atlantic Coast chiefly for Atlantic salmon and alosines; along Pacific Coast chiefly for salmon and trout). The dams are arranged approximately from north to south by region and were selected to include a range of sizes. Sources: Duda *et al.* (*189*), Brewitt (*190*) and multiple published sources. In some cases, the reported years of construction and removal may differ among sources, reflecting project’s whose initiation and completion spanned more than 1 year.

**Dam name**	**River**	**Region**	**Height (m)**	**Year Built**	**Removed**
		Atlantic Coast			
Veazie	Penobscot River	Maine	9.1	1913	2013
Edwards	Kennebec River	Maine	7.3	1837	1999
Smelt Hill	Presumpscot	Maine	4.3	1898	2002
Elm Street	Jones River	Cape Cod (Massachusetts)	2.7	1920	2019
Weston Mill Dam	Millstone River	New Jersey	1.5	1844	2017
Bloede	Patapsco River	Maryland	10.4	1906	2018
Embrey	Rappahannock River	Virginia	6.7	1855	2005
Milburnie	Neuse River	North Carolina	4.6	1813	2017
Conagree	Congaree	South Carolina	4.6	1950’s	2019
White	Oconee	Georgia	4.4	1912	2018
		Pacific Coast			
Elwha	Elwha River	Olympic Peninsula (Washington)	32	1913	2012
Glines Canyon	Elwha River	Olympic Peninsula (Washington)	64	1927	2014
Goldsborough Creek	Goldsborough Creek	Puget Sound (Washington)	10.7	1921	2001
Condit	White Salmon River	Columbia River (Washington)	38.1	1913	2012
Little Sandy	Little Sandy River	Columbia River (Oregon)	4.6	1913	2008
Gold Ray	Rogue River	Oregon coast	11.6	1941*	2010
Gold Hill diversion	Rogue River	Oregon coast	2.4	1931	2008
Sweasy	Mad River	California coast	16.8	1938	1970
York Creek diversion	York Creek	California coast	1.5	1900	2004
San Clemente	Carmel River	California coast	32.2	1921	2015

*For example, Gold Ray Dam was initially built as a wooden structure in 1904 and a concrete structure replaced it in 1941.

There are other options for improved fish passage besides dam removals, and all need to be considered where removals are not presently possible or desirable. Natural fishways, i.e., artificial channels routed around dams, are a particularly promising approach but their application is limited by slope and area. Some dams may become more passable by notching or lowering the distance between the reservoir and river below the dam. The body size, morphology, swimming performance, and behavior of diadromous fishes vary greatly, so modifications must be tailored to suit the species in question such as Pacific lamprey (*120*, *173*) or optimized for the community of species, including not only large, named dams but also the myriad small and less conspicuous culverts and other blockages.

In the United States, changes in how diadromous fish restorations are viewed could help encourage and foster more ecologically and societally meaningful recoveries. One is a greater focus on the watershed level. For American hydro-dams, the process of the Federal Energy Regulatory Commission allows for substantial discrepancies in timing for relicensing reviews among individual hydro-dams on a river. It would be helpful to conduct relicensing evaluations as concurrently as possible for the dams on a given river to encourage whole-watershed thinking, as exemplified by the creative solutions to maintain electrical generation while reopening many miles of river that occurred on the Penobscot River. Such thinking should include the development of resilience-building increases in spatial distribution and life history variation within river systems (*174*) through habitat restoration and reconnection (*175*). The choice should not be whether to remove passage barriers or improve accessible habitat, but both.

It is also valuable to preserve the few American rivers with little or no damming as protected areas (*176*) serving as reference systems (*1*) for the characteristics of healthy populations of diadromous fishes and as sources or seed populations for regional population complexes. In the U.S. Northeast, the mainstem Delaware River has no dams and, following pollution reductions, now has resurgent populations of American shad, striped bass, and Atlantic sturgeon. Concerns about salmon conservation are anything but new, and one idea is to give special protection to strongholds—regions with especially abundant, productive, and diverse populations. In the late 19th century, there was a call for a “National Salmon Park” to give Pacific salmon the kind of sanctuary that Yellowstone National Park provided for the American bison (*177*). Calls for such strongholds were made by former EPA Administrator William Ruckelshaus and Oregon governor John Kitzhaber who wrote, “…by protecting the best remaining salmon ecosystems throughout their range, wild salmon cannot only survive, but thrive, for generations to come” (*178*). The stronghold concept is prominent in the literature on trout conservation (*179*), although concerns for its practicality “…without more holistic and risk-averse management” (*180*) might limit its application.

It also is important in considering future restoration actions to take retrospective views of the diadromous fishes of a watershed. The success of fish restoration projects should be assessed not merely based on comparisons with the levels of abundance immediately before the project or minor increases in extremely low fish passage rates, but against a broader context of historical ecology that considers what an unhindered river produced in the more distant past and its broader societal benefits. This could include both a greater emphasis on historical information (*1*, *125*) and the modeling of carrying capacities under different management regimes, as conducted for Gulf sturgeon, *A. oxyrinchus desotoi* (*181*). Such efforts would push back against the shifting baselines syndrome (*182*) that may limit the visions of fish population managers and result in more ambitious numerical restoration targets.

The United States has more dams than any other nation (*183*), with detrimental consequences for diadromous fishes and many other aspects of river ecosystems. However, between 1968 and 2019, 1654 dams of all sizes were dismantled in the United States, with an increasing trend for dams less than 7.5 m high. If past trends continue, then there should be a slow amelioration of dam effects; forecasts are for between 4000 and 36,000 dam removals in the United States by 2050, and more dams are being removed in North America and Europe than are being built (*183*).

Vast amounts of money have been spent for the preservation of Atlantic and Pacific salmon and other diadromous fishes, often with little or no benefit to the fish. Meanwhile, populations of these fishes are at ever increasing risk of extinction. We have categorized their drivers of decline and found a range of remediation potential and effect that, in the future, will be largely ineffectual, ranging from those largely remediated, to those continuing to be remediated, to those typically irreversible, to those remediable in theory but not in practice. Then, there is that which is available for remediation: Removal of dams and other barriers has proven highly successful and could be increasingly implemented today. The future of American diadromous fishes is in the balance.
