# Supplementary material for: North American diadromous fishes: Drivers of decline and potential for recovery in the Anthropocene
Source: Sci Adv. 2022 Jan 28;8(4):eabl5486. doi: 10.1126/sciadv.abl5486 (PMC8797777; doi:10.1126/sciadv.abl5486)
Supplement: Supplementary file 1 — Legends for data files S1 and S2 [file sciadv.abl5486_sm.pdf]

Science Advances
AAAS

Supplementary Materials for

North American diadromous fishes: Drivers of decline and potential for recovery in the Anthropocene

John R. Waldman* and Thomas P. Quinn

*Corresponding author. Email: john.waldman@qc.cuny.edu

Published 28 January 2022, *Sci. Adv.* **8**, eabl5486 (2022)
DOI: 10.1126/sciadv.abl5486

The PDF file includes:

Legends for data files S1 and S2

Other Supplementary Material for this manuscript includes the following:

Data files S1 and S2

Captions for Data files (S1-S2)

Supplementary Data S1

Name of dam, dimensions of dam, river of occurrence, year of completion, year of removal, location, descriptive publication, and additional ancillary information.

Supplementary Data S2

Name of dam, dimensions and materials of dam, river of occurrence, original purpose, year of completion, year of removal, location, and additional ancillary information.
